# Effects of Protein Source, Whole Wheat and Butyric Acid on Live Performance, Gut Health and Amino Acid Digestibility in Broiler Chickens

**DOI:** 10.3390/metabo12100989

**Published:** 2022-10-19

**Authors:** Shafqat N. Qaisrani, Ali I. Hussain, Saima Naveed, Fehmeada Bibi, Chaudhry A. Akram, Talat N. Pasha, Muhammad Asif, Irfan Irshad, Rana M. Bilal

**Affiliations:** 1 Department of Animal Nutrition, Faculty of Animal Production & Technology, University of Veterinary & Animal Sciences, Lahore 54000, Pakistan; 2 Department of Zoology, University of Education Lahore, Multan Campus, Bosan Road, Multan 60000, Pakistan; 3 Faculty of Veterinary Sciences, Bahauddin Zakariya University, Multan 60800, Pakistan; 4 Township Campus, University of Education Lahore, Lahore 54770, Pakistan; 5 Institute of Continuing Education & Extension, University of Veterinary & Animal Sciences, Lahore 54000, Pakistan; 6 Department of Animal Nutrition, Faculty of Veterinary & Animal Sciences, The Islamia University of Bahawalpur, Bahawalpur 63100, Pakistan

**Keywords:** broiler, whole wheat, butyric acid, growth performance, gut health, amino acid digestibility

## Abstract

A total of 896 1-day-old straight-run (Ross-308) broilers were used to investigate the interactive effects of protein source (PS), diet structure (DS) and butyric acid (BA) on live performance and carcass characteristics, gut development and its morphology and apparent ileal digestibility (AID) of protein and amino acids (AA). Eight experimental diets comprising 8 replicates with 14 birds each were tested in a 2 × 2 × 2 factorial arrangement with complete randomized design by two levels of BA (0 and 0.1%), two forms of DS (whole vs. ground wheat) and two PS, i.e., soybean meal and canola meal (SBM vs. CM). Throughout the entire experimental period (0 to 35 d), broilers fed SBM-based diets exhibited better (*p* < 0.05) growth performance (feed intake (FI), body weight gain (BWG) and feed conversion ratio (FCR)), carcass parameters (*p* < 0.05), gut health (*p* < 0.05), and nutrient digestibility (*p* < 0.05) than CM-fed broilers. Dietary whole wheat (WW) positively affected FI (*p* = 0.001), BWG (*p* = 0.004) and FCR (*p* = 0.035) during the overall experimental period. Broilers fed WW had 6, 5, 8, 11 and 10% lower empty relative weights of crop, proventriculus, jejunum, ileum and colon and 25 and 15% heavier gizzard and pancreas, respectively, with longer villus height (*p* < 0.001), reduced crypt depth (*p* = 0.031) and longer villus height-to-crypt depth ratio (*p* < 0.001) than those fed ground-wheat-based diets. Broilers fed WW had greater (*p* < 0.05) AID of CP and most of the AA. Butyric acid supplementation resulted in improved (*p* < 0.05) growth performance and digestibility of threonine, valine, leucine, isoleucine, phenylalanine, serine and aspartate. The broilers consuming SBM had 28% lower abdominal fat than those fed CM-based diets. In conclusion, harmful consequences of a less digestible PS can partially be compensated by the inclusion of WW, and supplementation of BA further reduces these detrimental effects.

## 1. Introduction

Protein (amino acids) is one of the expensive components of poultry ration. The efficiency of a protein feedstuff for poultry depends on its ability to provide a sufficient amount of essential amino acid prerequisite to the bird, as modern-day broilers are more sensitive to dietary protein (amino acids) than energy [1]. Sustainable, efficient and ecofriendly poultry production requires highly digestible amino acids, resulting in an unremitting competition amongst poultry and humans for protein sources, including soybean meal (SBM). This competition leads to increased costs of SBM, creating a simulated food shortage among the poorest societies worldwide. Canola meal (CM) is the most commonly used plant protein source in poultry feed following SBM [2], with lower CP than SBM (38 vs. 48%), although it is comparable with SBM in terms of amino acid content expressed per 100 g of CP. Canola meal is also a quality source of sulfur-containing amino acids, such as methionine + cystine (0.74 vs. 0.65), with a higher fat content than SBM (3.6 vs. 1.3% of DM) due to presence of triglycerides, phospholipids, glycolipids and free fatty acids. However, the CP contents in CM are less digestible (62%) than those in SBM (72%) [2]. There is a limited use of CM in poultry feed, owing to its less metabolizable energy (2000 vs. 2230 kcal/kg) and the presence of various antinutritional factors containing fiber, glucosinolates, phytate, sinapine, non-starch polysaccharides and tannins [3,4] that may reduce the digestibility of AA and energy, leading to poor growth performance.

Whole wheat (WW) feeding in poultry, a common practice in wheat-dominant producing countries, can be an effective nutritional tool to enhance the availability of nutrients in low-digestible protein sources, including CM. These whole grains are crushed into small particles in the gizzard, resulting in a longer retention time leading to an increased gizzard weight as a result of prolonged mechanical stimulation [5]. A well-functioning gizzard increases gut motility by enhancing cholecystokinin secretion [6], which can induce the excretion of pancreatic enzymes and gastro-duodenal refluxes [7]. This prolonged retention time in the gizzard consequently increases the efficacy of digestive enzymes and HCl released from the proventriculus into the gizzard [5], which promotes efficient nutrient digestion and absorption, resulting in an improved growth performance in broilers.

Supplementation of organic acids, including butyric acid (BA), as a feed additive is an alternative way to enhance the availability of nutrients in broilers [8]. The BA stimulates the multiplication and differentiation of gut epithelial cells, as it is an easily accessible energy source for these cells, improving mucosal immune response by increasing the growth of gut-associated lymphoid tissue and anti-inflammatory impact and reducing the detrimental microbiota population in the gut [9,10,11,12]. Supplementation of BA enhances functional growth of the gut due to an improved villus height (VH) and reduced crypt depth (CD), leading to increased availability of nutrients in broilers [13]. This increased VH results in an increased surface area for nutrient absorption in the small intestine and improved nutrient utilization, resulting in better live performance of broilers [11,12].

It was postulated that BA supplementation, along with WW, in a less digestible protein source-based diet, may counterbalance the negative consequences of the protein source with respect to overall growth performance of broilers. Therefore, the present study was executed to explore the interacting impact of protein source (PS), diet structure (DS) and BA supplementation on live performance, carcass characteristics, gut development and health and apparent ileal digestibility (AID) of nutrients (CP and AA) in broilers.

## 2. Materials and Methods

### 2.1. Birds and Experimental Diets

A total of 896 1-d-old straight-run broilers were arbitrarily distributed across 64 floor pens (1.6 × 1.3 × 0.5 m^3^; L × W × H) in an environmentally controlled room. A completely randomized design with a factorial arrangement of 2 × 2 × 2 including 8 treatments with 8 replicates comprising 14 birds each was used to evaluate the main and interaction impacts of diet structure (whole vs. ground wheat), protein source (SBM vs. CM) and BA supplementation (yes vs. no) in the broilers. Before placement in the pens, chicks were weighed to ensure consistency in the pen weights. Each pen had a feeding trough and four nipple drinkers. Fresh rice husk was used as a litter material during the trial. A whole wheat diet was produced by adding WW directly in a mixer before pelleting, whereas the wheat grains were ground in a hammer mill for the ground-wheat-based diets. Butyric acid (calcium butyrate, Impex international, Lahore, Pakistan) was supplemented at a rate of 0.1% in the feed. Table 1 shows the formulated feed according to the strain recommendations (Ross-308), which was offered as 2.5 mm pellets for the starter (0 to 14 days) and 4 mm pellets for the grower (15 to 35 days) period. Neither antimicrobial growth promoters nor coccidiostats were supplemented in the diets.

### 2.2. Experimental Procedure and Sampling

The experimental protocols were conducted in accordance the Animal Ethics Commission recommendations of the University of Veterinary and Animal Sciences, Lahore, Pakistan. The room temperature was kept at 33 °C for the first three days and, thereafter, steadily reduced by 3 °C per wk to a constant value of 24 °C at day 21. During the first three days, a lightning period of 23 h per day was set to facilitate continuous feed and water intake, and a 16 h per day light period was maintained subsequently throughout the trial, with fluorescent tubes used as a light source. Water was available *ad libitum* until slaughtering. For the digestibility trial, 2% Celite^®^ was added as an inert marker in the feed during the last three days of the trial. The broilers were monitored twice a day during the trial for welfare issues, including wounds and general health.

Mortality was documented when it occurred. Feed intake per pen was noted weekly by subtracting the leftover (g) in the feed trough from the total feed offered. Body weight gain per week was computed by subtracting the weight of the pen recorded last week from the weight of each pen at the end of that week. FCR was evaluated by dividing total FI by weight gain of live plus dead birds. On day 36th, four birds per replicate were randomly slaughtered, and their abdominal cavities opened. The various sections of the gastrointestinal tract including, crop, proventriculus, gizzard, duodenum, jejunum and ileum, were segmented, digesta were removed and the empty segments were weighed immediately. The contents of the terminal ileum were cumulated by moderate flushing with distilled water into a plastic bottle. To avoid contamination, the terminal ileum, a section of the GIT from 15 to 2 cm anterior to the ileo-cecal junction, was trimmed. The ileal digesta of the birds in a pen was pooled, yielding eight composite samples per dietary treatment, and immediately frozen after collection. The digesta samples were ground to pass through a 0.5 mm sieve and stored in airtight containers at −4 °C for chemical analysis. Duodenal sections (about 2 cm long) from the midsection of the duodenum were taken for morphological analysis, rinsed with 0.9% cold physiological saline and immediately placed in Bouin’s fluid. The samples were immersed in 70% ethanol, thereafter, for 24 h before being fixed in paraffin and sectioned with 5 µm thickness.

Each slaughtered bird was defeathered to determine carcass weight. Following elimination of the head, paws, abdominal fat and giblets, the eviscerated weight was measured. Weighing was done for the breast meat yield (with pectoralis major and minor muscles), leg quarter (with thigh and drumstick muscles) and abdominal fat (with leaf fat adjoining the cloaca and abdominal fat adjoining the gizzard).

### 2.3. Analytical Procedures

For morphometric analysis, six duodenal cross sections from each broiler were prepared according to hematoxylin and eosin procedure [14]. Villus height (the distance between the top of the villus and the juncture between the villus and the crypt), crypt depth (the space between the juncture and the basement membrane of the epithelial cells at the base of the crypt) and villus height to crypt depth ratio (VCR) were measured on 10 integral, well-oriented villi (from 2 cm in the midsection of the duodenum) of each bird by a compound light microscope furnished with a video camera.

To determine the apparent ileal digestibility of nutrients (CP and AA), the stored feed and digesta samples were dried and ground using a 0.5 mm screen fitted in a variable-speed rotor mill (PULVERISETTE 14). These ground samples were used to determine acid insoluble ash (AIA) and N contents (AOAC, 2000) [15]. The AA contents in the diets and the ileal contents were evaluated by ion-exchange chromatography. Samples were prepared for oxidation and hydrolysis [16]. Briefly, the samples were oxidized with performic acid (Merck KGaA 64271 Darmstadt, Germany) and hydrolyzed by 6 M HCl with phenol (1 g/L) for 24 h at 110 °C. Thereafter, the hydrolysate was diluted, and the pH was adjusted to 2.2 by 7.5 M NaOH. The hydrolysates were passed through a sterilized syringe filter with a pore size of 0.2 µm (Filter Bio^®^ Top PES Sterile Syringe Filter). Then, the hydrolysates aliquots were exposed to ion-exchange chromatography with a Biochrom 30^+^ amino acid analyzer (Biochrom 30^+^, Biochrom Limited. Cambridge, UK).

The AID of CP and AA was calculated as described by [17].

$$\mathrm{AID}\;\mathrm{of}\;\mathrm{Nutrient}\;\left(\%\right)=\frac{\left(\mathrm{Nutrient}/\mathrm{AIA}\right)\;d\;-\;\left(\mathrm{Nutrient}/\mathrm{AIA}\right)\;i}{\left(\mathrm{Nutrient}/\mathrm{AIA}\right)\;d}\;\times 100$$

where (Nutrient/AIA) d is the ratio of nutrient (CP and AA) and AIA in the diet, and (Nutrient/AIA) i is the ratio of nutrient (CP and AA) and AIA in the ileal digesta.

### 2.4. Data Analysis

For data analysis, SAS PROC MIXED (version 9.1; SAS Inst. Inc., Cary, NC, USA) repeated statements were used with a generalized linear model. The data analysis was performed according to the following statistical model:

Y_ijkl_ = µ + PS_i_ + DS_j_ + BA_k_ + PS_i_ × DS_j_ + PS_i_ × BA_k_ + DS_j_ × BA_k_ + PS_i_ × DS_j_ × BA_k_ + e_ijkl_

Differences between treatments were considered significant at a probability level of 5% or lower.

## 3. Results

### 3.1. Live Performance

The impact of experimental diets on live performance in broilers is indicated in Table 2. In the starter phase (0 to 14 days), the dietary treatments did not influence FI, whereas BWG was influenced by PS (*p* = 0.001) and DS (*p* = 0.016), and FCR was influenced by PS (*p* = 0.016). During the mentioned period, the broilers fed SBM diets showed 20% higher BWG and 5% better FCR than those consuming CM. Whole wheat feeding resulted in 13% increased BWG relative to broilers consuming GW-based diets. The interactions between PS and DS were noted for FI (*p* = 0.032), BWG (*p* = 0.012) and FCR (*p* = 0.030) during the overall trial period (0 to 35 days of age), revealing that the broilers fed WW in CM diets showed improved FI and BWG, with better FCR, whereas WW in SBM diets did not affect the live performance (FI, BWG and FCR) throughout the experimental period. Interactions between PS and BA were noted for FI (*p* = 0.012) and FCR (*p* = 0.022), showing that broilers fed BA in CM based diets had higher FI with improved FCR, whereas BA supplementation in SBM diets had no influence on the stated parameters. Interactions between DS and BA were noted regarding FI (*p* = 0.034), BWG (*p* = 0.042) and FCR (*p* = 0.031), showing that the broilers consuming BA in GW based diets had improved FI and BWG with a better FCR, whereas BA in WW-based diets had no effect on live performance (FI, BWG and FCR).

### 3.2. Gut Development

The influence of experimental diets on empty relative weights of different intestinal parts and associated organs in the broilers is presented in Table 3. Broilers fed a WW based had, on an average, 6, 5, 8, 10 and 11% lower empty relative weights of crop, proventriculus, jejunum, ileum and colon, respectively, than those fed GW diets, irrespective of the PS. In contrast, the broilers fed WW diets, had a 25% heavier gizzard than those fed GW diets. An interaction between PS and DS was detected for the empty relative weights of the ceca (*p* = 0.041), showing that broilers fed GW based diets showed significantly higher empty relative cecal weight compared with those fed WW-based diets, irrespective of the PS.

### 3.3. Gizzard pH and Gut Morphology

Gizzard pH remained unaffected (*p* = 0.753) by PS (Table 4). Crypt depth was influenced by PS (*p* = 0.009), DS (*p* = 0.031) and BA (*p* = 0.002) supplementation. Broilers fed SBM had 25% lower CD and about 40% improved VCR relative to those fed CM based diets. Similarly, WW resulted in 8% and BA supplementation led to 16% lower CD relative to broilers fed GW and diets without BA supplementation. Significant interactions were observed for VH (*p* = 0.011) between PS and DS, revealing that WW based diets resulted in improved VH, irrespective of the PS offered to the broilers. Similarly, an interaction between PS and BA was noted for VH (*p* = 0.001), indicating increased VH in the broilers reared on BA supplemented CM based diets, whereas BA supplemented SBM did not alter the VH. Irrespective of the PS, there was a significant interaction between DS and BA with respect to gizzard pH (*p* = 0.023), VH (*p* = 0.006) and VCR (*p* = 0.012), showing that decreased gizzard pH increased VH with improved VCR in broilers fed GW based BA supplemented diets, whereas BA supplementation in WW based diets did not significantly affect the mentioned parameters.

### 3.4. Apparent Ileal Digestibility of Protein and Amino Acids

Effects of experimental diets on AID of CP and amino acid AID in broilers are shown in Table 5. Interactions between PS and DS were observed for digestibility of CP (*p* = 0.044), as well as Meth (*p* = 0.043) and Lys (*p* = 0.031), indicating that WW in CM containing diets resulted in an improved AID of CP, Meth, Cys and Lys, whereas WW did not influence this parameter in the broilers consuming SBM. Similarly, an interaction between PS and BA showed that BA supplementation improved the AID of CP in broilers consuming CM-based diets, whereas it remained unaffected in broilers consuming SBM. The broilers consuming an SBM diet exhibited improved (*p* < 0.05) digestibility of almost all amino acids, excluding histidine (*p* = 0.453) and glutamate (*p* = 0.521). Similarly, whole wheat feeding resulted in improved (*p* < 0.05) digestibility of cysteine, threonine, valine, leucine, isoleucine, phenylalanine, serine and aspartate, whereas the digestibility of arginine, histidine, glutamate, glycine, alanine and tyrosine remained unaffected (*p* < 0.05) compared with broilers fed GW-based diets.

### 3.5. Carcass Characteristics

The effect of dietary treatments on carcass parameters in broilers are shown in Table 6. Interactions were found for LW (*p* = 0.001), CW (*p* = 0.001), CY (*p* = 0.032), LQY (*p* < 0.001) and BY (*p* = 0.041) between PS and DS, indicating that broilers reared on WW CM based diets showed increased LW, CW, CY, LQY and BY, whereas WW, along with SBM, did not influence these parameters.

Interactions were detected between PS and BA for LW (*p* = 0.041), CW (*p* = 0.012) and BY (*p* = 0.037), showing that BA in CM-based diets resulted in improved LW, CW and BY, whereas this supplementation in SBM did not affect LW, CW or BY. An interaction between DS and BA was found for LW (*p* = 0.032), CW (*p* = 0.024), CY (*p* = 0.037), LQY (*p* = 0.045), BY (*p* = 0.030) and ABF (*p* = 0.043), demonstrating that broilers consuming BA in GW-containing diets exhibited increased LW, CW, CY, LQY and BY and decreased ABF, whereas BA-supplemented WW-based diets did not influence LW, CW, CY, LQY, BY or ABF, irrespective of the PS. Abdominal fat contents were influenced by PS (*p* = 0.015). The broilers consuming SBM-containing diets had 28% lower abdominal fat than those consuming CM.

## 4. Discussion

The present study was executed to analyze the interacting effects of PS, DS and BA supplementation on live performance, carcass characteristics, gut development and health and AID of protein and AA in broilers. We hypothesized that a WW based diet supplemented with BA may counterbalance the negative consequences of poorly digestible protein sources, including CM, on growth performance in broilers.

The reduced live performance (lower FI, reduced BWG and poorer FCR) and a decreased carcass yield in the broilers fed CM diets relative to those consuming SBM diets are in agreement with the results of previous studies reported in broilers [14,18,19]. The observed compromised live performance in broilers consuming diets with increasing levels of CM may be related to various antinutritional factors present in CM, including tannins, glucosinolates, sinapine, NSPs and phytate [20]. These antinutritional factors, including tannins and phytic acid, make protein–enzyme complexes in the GIT, resulting in a reduction in protein digestion [2,18]. This diminished digestion may lead to a reduced absorption and eventually compromised growth performance, which may also be explained by a poorer gut health (shorter villi, deeper crypts and reduced VCR; Table 4) than broilers fed SBM based diet. This poorer gut health in broilers fed CM diets is in accordance with the literature on broilers [14,19], leading to reduced availability of protein and amino acids (Table 4 and Table 5). Longer villi and shorter crypts are associated with increased absorption of nutrients in the small intestine. Live performance results reported in this study also support the existence of shorter villi in broilers fed CM diets. Furthermore, extra maintenance energy and nutrients, are required for gut repair and liver metabolic functions when broilers are fed diets containing high levels (>20%) of CM, resulting in a reduced growth performance.

Poor gut health and reduced digestibility of nutrients (CP and AA) in CM consuming broilers indicate a decreased relative yield of carcass relative to those fed SBM diets, which corresponds to the results of previous research [19,21,22]. Breast meat yield is believed to be hyper-sensitive to the amount and composition of dietary amino acids [22]. A reduced digestibility of most AAs, including lysine, methionine, cysteine, threonine, arginine and glutamic acid, in CM fed broilers therefore led to diminished carcass characteristics, especially the breast muscles, because lysine is essential for the growth of the pectoralis major muscle (a breast muscle composed entirely of fast-twitch glycolytic fibers) [23]. Similarly, via the formation of glutamate, arginine proliferates the quantities of proline and hydroxyproline that are essential for connective tissue synthesis [24].

The improved growth performance (FI, BWG and FCR) in the broilers fed WW based diets compared to those fed GW based diets can be attributed to a well-functioning gizzard, which enhances gut motility, in particular, gastro-duodenal reflux [7]. This reflux, along with a reduced passage rate of digesta, results in a prolonged time budget available for mixing of enzymes with feed particles in the gut. The reduced gizzard pH in broilers consuming WW relative to those fed GW may be explained by chyme reflux between the proventriculus and gizzard and increased HCl production. Pancreatic enzymes more efficiently denature and hydrolyze dietary protein in this low-pH environment, resulting in an enhanced protein digestion [25,26], which is confirmed by outcomes reported in the present study.

Lower crop and proventriculus relative empty weights in WW fed broilers may be a result of the short residence time of the feed in these organs relative to broilers consuming GW diets because a well-functioning gizzard acts as a FI controller and prohibits overconsumption of feed by vagal signals activated by stretch and muscular movement [27] or by humoral signals comprising cholecystokinin, ghrelin and gastrin [28]. This improvement in the gizzard weights may be the result of larger particle size of the diet, resulting in increased mechanical stimulations of the gizzard muscles, which lead to improved enzyme–substrate interaction, resulting in an improved nutrient digestion and gut health [5,29], as indicated by improved digestion of proteins and amino acids (Table 4 and Table 5) and improved gut health data (Table 4). A higher relative weight of the pancreas may indicate increased pancreatic secretion in WW fed broilers relative to those fed GW based diets. The lower relative empty weights of the duodenums, jejunums and ileum in WW fed broilers can be attributed by their reduced activity due to a well-developed and functional gizzard, resulting in an appropriate pre-digestion in the foregut [25]. Improved digestibility of most of AAs, including threonine, may also lead to improve gut health because higher digestibility results in an increased availability of threonine in the gut. This greater availability of threonine, for instance, stimulates mucin synthesis, leading to improved intestinal defense, its healing with an improved gut health [30]. Threonine is a component of mucin, which accounts for approximately 40% of total digestive tract proteins [31], whereas mucin itself contains about 30% threonine [32], which protects the intestine from chemical secretions and pathogens, leading to an improved gut morphology.

Improved carcass yield in broilers consuming WW relative to those fed GW can be attributed to improved gut health, resulting improved nutrient digestion, especially protein and amino acids. A healthy and well-functioning gut may require fewer nutrients (protein and energy) for its maintenance and; therefore, more nutrients will be available for growth and carcass development. Decreased abdominal fat weight in broilers fed WW diets is in agreement with the results of a previous study of broilers [33]. The improved digestibility of lysine and threonine also supports improved carcass characteristics in broilers fed WW based diets because the mentioned AAs are responsible for muscle development in broilers [23,24].

The improved live performance in the broilers consuming BA supplemented diets is in agreement with previously published data on broilers [11,12,13,14]. Supplementation of 0.004 g/g of BA glycerides resulted in an 8% improvement in BW and a 6% in FCR relative to broilers consuming diets without BA [34]. Qaisrani et al. [14] reported 7 and 4% improved BWG and 5 and 3% improved FCR in starter and grower phases, respectively, in broilers consuming diets with BA. The reduction in gizzard pH in broilers fed BA diets could be the result of BA absorption in the foregut, as about 60% of the BA is absorbed in this organ [35]. The improved nutrients digestion and absorption in broilers fed diets with BA observed in the present study can be attributed to reduced gizzard pH, increased pancreatic secretion and the positive influence of BA on gut mucosal integrity and repair; furthermore, antimicrobial activity may have led to an improved growth performance. Improved gut health (increased VH, decreased CD and enhanced VCR) in broilers fed diets with BA could be the result of the availability of energy to enterocytes, as BA is the main energy source for these cells [5]. The improved VH and VCR and decreased CD in broilers reared on BA supplemented diets are in line with literature reports of broilers [11,13]. Increased CD may have a deleterious impact on the performance of birds, as it increases the mucosa turnover rate, which is involved in increasing maintenance requirements in broilers [36]. Morphometric changes in the gut as a result of BA supplementation extends the surface area for nutrient penetration, leading to enhanced growth performance, as confirmed by the data (Table 2). As expected, BA supplementation enhanced the AID of protein and AAs in broilers relative to those fed diets without BA, in agreement with previous studies of broilers [13,37,38]. Increased AA and protein digestibility may be the result of improved gut health (increased VH and VCR and reduced CD) in broilers fed BA supplemented diets relative to those fed diets without BA.

Decreased duodenal weight in broilers consuming diets with BA may be the result of reduced activity due to a functional muscular gizzard, which ensures maximum digestion, resulting in less work available for duodenum, leading to its decreased weight. The improved breast meat yield in broilers fed BA supplemented diets may be the result of improved ileal digestibility of nutrients (CP and AA), especially lysine and threonine, as discussed above. Lysine is the most critical essential amino acid involved in protein synthesis and breast muscle deposition in broilers. Nasr and Kheiri [39] reported a 14% increased breast meat yield in broilers fed 10% extra dietary lysine relative to those consuming diets with NRC-endorsed dietary levels of lysine.

The results of the present study suggest that CM can lead to diminished live performance, compromised gut morphology and reduced AID of CP and AA. In contrasts, the inclusion of WW in broiler diets, resulted in a heavier gizzard, improved gut development and morphology and better AID of CP and AA, leading to improved FCR and carcass characteristics. Furthermore, supplementation of BA has additional positive impacts on overall performance of broilers fed WW based diets. In conclusion, WW based diets with BA enhanced the live performance of broilers consuming even less digestible protein source (CM). 

## Figures and Tables

**Table 1:** Ingredients and nutrient contents of the experimental diets (as-fed basis).

Ingredients (%)	Soybean Meal		Canola Meal	
	Starter	Grower	Starter	Grower
Corn	50.5	54.8	49.5	52.1
Wheat	10.0	10.0	10.0	10.0
Soybean meal (46%)	28.0	25.0	-	-
Canola meal (36%)	-	-	25.0	22.0
Poultry byproduct meal	5.0	3.0	8.0	8.0
Vegetable oil	2.0	3.0	2.5	3.5
CaCO_3_	1.3	1.2	1	0.92
MCP	1.4	1.3	1.3	1.1
Premix ^1^	0.5	0.5	0.5	0.5
L-HCL	0.45	0.43	0.85	0.73
DLM	0.36	0.34	0.34	0.3
Threonine	0.16	0.16	0.3	0.21
NACL	0.14	0.1	-	-
NAHCO3	0.19	0.19	0.2	0.19
Tryptophan	-	-	0.05	0.05
L-Arginine	-	-	0.44	0.39
Total	100	100	100	100
Calculated nutrient levels (%)				
ME (kcal/kg)	3000	3100	3000	3090
DM	90	90	90	90
CP	22	20	21.88	19.93
CF	3.8	4.6	4.52	4.22
Calcium	0.96	0.86	0.96	0.86
Av. P	0.48	0.43	0.48	0.43
D-Lysine	1.28	1.16	1.28	1.16
D-Threonine	0.86	0.77	0.86	0.77
D-Tryptophan	0.20	0.18	0.20	0.18
D-M+C	0.95	0.87	0.95	0.87
D-Arginine	1.37	1.23	1.37	1.23
D-Valine	0.96	0.87	0.96	0.87
D-Isoleucine	0.86	0.78	0.86	0.78
D-Leucine	1.41	1.27	1.41	1.27

^1^ Premix composition (per kg of diet): 1.5 mg menadione, 2400 IE cholecalciferol, 7.5 mg riboflavin, 50 mg dl-a-tocopherol, 3.5 mg pyridoxine, 12,000 IE retinol, 36 mg niacin, 2.0 mg thiamine, 19 mg cyanocobalamins, 0.2 mg biotin, 11 mg D-pantothenic acid, 0.8 mg iodine, 81 mg iron, 85 mg manganese, 12 mg copper, 0.43 mg cobalt, 62 mg zinc, 460 mg choline chloride, 0.1 mg selenium, 1.0 mg folic acid, 125 mg anti-oxidant mixture.

**Table 2:** Effects of protein source (PS), diet structure (DS) and butyric acid (BA) supplementation on feed intake (FI), body weight gain (BWG) and feed conversion ratio (FCR) in broilers ^1^.

Effect	Observations					
	FI (g/bird/d)		BW Gain (g/bird/d)		FCR (g/g)	
Age (d)	0–14	0–35	0–14	0–35	0–14	0–35
SBM						
Ground						
With BA	28.4	106.2 ^a^	23.5 ^b^	67.6 ^ab^	1.21 ^a^	1.58 ^ab^
Without BA	26.7	103.3 ^b^	21.7 ^b^	64.7 ^b^	1.23 ^ab^	1.60 ^bc^
Whole						
With BA	31.9	108.3 ^a^	26.7 ^a^	70.5 ^a^	1.19 ^a^	1.53 ^a^
Without BA	30.4	106.1 ^a^	25.4 ^a^	68.2 ^a^	1.20 ^a^	1.55 ^a^
CM						
Ground						
With BA	24.4	98.3 ^c^	19.5 ^c^	58.3 ^c^	1.25 ^b^	1.69 ^e^
Without BA	23.8	94.8 ^d^	18.8 ^c^	53.2 ^d^	1.27 ^b^	1.78 ^f^
Whole						
With BA	26.5	100.6 ^c^	21.7 ^b^	62.1 ^b^	1.22 ^ab^	1.62 ^bc^
Without BA	26.1	98.1 ^c^	20.8 ^bc^	59.9 ^bc^	1.25 ^b^	1.64 ^cd^
Pooled SE	0.86	1.04	0.78	2.02	0.02	0.02
*p*-value						
PS	0.23	0.001	0.001	0.002	0.016	0.013
DS	0.35	0.001	0.016	0.004	0.065	0.035
BA	0.45	0.023	0.381	0.003	0.071	0.008
PS × DS	0.56	0.032	0.152	0.012	0.401	0.030
PS × BA	0.24	0.012	0.190	0.583	0.654	0.022
DS × BA	0.37	0.034	0.212	0.042	0.432	0.031

^a–f^ Means without a common superscript within a column differ significantly (*p* < 0.05). ^1^ Each value represents the mean of eight replicates (14 birds per replicate).

**Table 3:** Effect of protein source (PS), diet structure (DS) and butyric acid (BA) supplementation on empty relative weight (g/100 g of BW) of different intestinal segments and associated organs in broilers ^1^ at 36 days of age.

Effect	Crop	Proventriculus	Gizzard	Pancreas	Duodenum	Jejunum	Ileum	Ceca	Colon
SBM									
Ground									
With BA	0.40 ^a^	0.69 ^a^	1.10 ^b^	0.27 ^b^	0.90 ^c^	1.31 ^a^	1.21 ^b^	0.36 ^b^	0.20 ^b^
Without BA	0.38 ^a^	0.67 ^ab^	1.03 ^c^	0.26 ^b^	0.92 ^c^	1.34 ^a^	1.19 ^b^	0.35 ^b^	0.21 ^a^
Whole									
With BA	0.35 ^b^	0.64 ^b^	1.31 ^a^	0.31 ^a^	0.75 ^d^	1.22 ^b^	1.10 ^cd^	0.31 ^d^	0.18 ^b^
Without BA	0.36 ^b^	0.64 ^b^	1.29 ^a^	0.30 ^a^	0.78 ^d^	1.24 ^b^	1.11 ^cd^	0.32 ^cd^	0.19 ^b^
CM									
Ground									
With BA	0.39 ^a^	0.67 ^ab^	1.15 ^b^	0.26 ^b^	0.97 ^b^	1.33 ^a^	1.27 ^a^	0.40 ^a^	0.22 ^a^
Without BA	0.38 ^a^	0.65 ^b^	1.06 ^c^	0.26 ^b^	1.05 ^a^	1.35 ^a^	1.26 ^a^	0.39 ^a^	0.23 ^a^
Whole									
With BA	0.34 ^b^	0.63 ^c^	1.30 ^a^	0.32 ^a^	0.76 ^d^	1.21 ^b^	1.13 ^c^	0.32 ^cd^	0.18 ^b^
Without BA	0.35 ^b^	0.61 ^c^	1.28 ^a^	0.30 ^a^	0.79 ^d^	1.23 ^b^	1.15 ^bc^	0.34 ^c^	0.19 ^b^
Pooled SE	0.01	0.01	0.02	0.01	0.02	0.02	0.02	0.01	0.01
*p*-value									
PS	0.294	0.61	0.536	0.431	0.001	0.721	0.122	0.067	0.431
DS	0.001	0.003	0.001	0.021	0.003	0.002	0.031	0.024	0.041
BA	0.423	0.281	0.041	0.372	0.016	0.755	0.728	0.538	0.342
PS × DS	0.354	0.432	0.064	0.257	0.231	0.112	0.413	0.041	0.158
PS × BA	0.468	0.354	0.378	0.314	0.721	0.253	0.390	0.322	0.234
DS × BA	0.531	0.062	0.071	0.124	0.122	0.260	0.598	0.427	0.281

^a–d^ Means without a common superscript within a column differ significantly (*p* < 0.05). ^1^ Each value represents the mean of eight replicates (four birds per replicate).

**Table 4:** Effect of protein source (PS), diet structure (DS) and butyric acid (BA) supplementation on gizzard pH, villus height (VH), crypt depth (CD) and villus height-to-crypt depth ratio (VCR) in broilers ^1^ at 36 days of age.

Effect	Gizzard pH	VH (µm)	CD (µm)	VCR
SBM				
Ground				
With BA	2.6 ^b^	1523 ^c^	172 ^d^	8.85 ^c^
Without BA	3.1 ^a^	1490 ^d^	193 ^c^	7.72 ^d^
Whole				
With BA	2.0 ^c^	1680 ^a^	152 ^e^	11.1 ^a^
Without BA	2.4 ^c^	1610 ^ab^	167 ^d^	9.64 ^b^
CM				
Ground				
With BA	2.7 ^b^	1253 ^f^	210 ^b^	5.97 ^f^
Without BA	3.2 ^a^	1105 ^g^	254 ^a^	4.35 ^g^
Whole				
With BA	2.1 ^c^	1360 ^e^	203 ^b^	6.70 ^e^
Without BA	2.3 ^c^	1283 ^ef^	244 ^a^	5.26 ^f^
Pooled SE	0.01	45.4	4.33	0.21
*p*-value				
PS	0.753	<0.001	0.009	<0.001
DS	0.001	<0.001	0.031	<0.001
BA	0.045	<0.001	0.002	<0.001
PS × DS	0.154	0.011	0.844	0.169
PS × BA	0.532	0.001	0.112	0.568
DS × BA	0.023	0.006	0.839	0.012

^a–g^ Means without a common superscript within a column differ significantly (*p* < 0.05). ^1^ Each value represents the mean of eight replicates (four birds per replicate).

**Table 5:** Effect of protein source (PS), diet structure (DS) and butyric acid (BA) supplementation on apparent ileal digestibility of protein and amino acids in broilers ^1^ at 36 days of age.

Effect	CP	Meth	Cys	Lys	Thr	Val	Arg	Leu	Iso	Phe	Hist	Asp	Ser	Glu	Gly	Ala	Tyr
SBM	0.75 ^x^	0.92 ^x^	0.75 ^x^	0.88 ^x^	0.83 ^x^	0.83 ^x^	0.88 ^x^	0.89 ^x^	0.88 ^x^	0.87 ^x^	0.88	0.85 ^x^	0.86 ^x^	0.90	0.84 ^x^	0.86 ^x^	0.88 ^x^
Ground																	
With BA	0.74 ^b^	0.91 ^a^	0.74	0.88 ^b^	0.82 ^ab^	0.81 ^ab^	0.86	0.86 ^b^	0.85 ^b^	0.84 ^b^	0.87	0.83 ^b^	0.84 ^b^	0.88	0.84	0.85	0.88
Without BA	0.73 ^bc^	0.90 ^ab^	0.74	0.86 ^b^	0.81 ^b^	0.80 ^b^	0.85	0.85 ^b^	0.83 ^b^	0.83 ^b^	0.87	0.81 ^b^	0.82 ^b^	0.88	0.83	0.84	0.86
Whole																	
With BA	0.76 ^a^	0.93 ^a^	0.76	0.91 ^a^	0.86 ^a^	0.86 ^a^	0.92	0.91 ^a^	0.92 ^a^	0.89 ^a^	0.89	0.88 ^a^	0.89 ^a^	0.91	0.85	0.86	0.89
Without BA	0.74 ^ab^	0.92 ^a^	0.75	0.90 ^a^	0.83 ^a^	0.83 ^a^	0.89	0.89 ^a^	0.90 ^a^	0.88 ^a^	0.88	0.87 ^a^	0.88 ^a^	0.90	0.84	0.85	0.88
CM	0.65 ^y^	0.87 ^y^	0.68 ^y^	0.79 ^y^	0.71 ^y^	0.77 ^y^	0.79 ^y^	0.81 ^y^	0.77 ^y^	0.79 ^y^	0.84	0.76 ^y^	0.75 ^y^	0.86	0.77 ^y^	0.79 ^y^	0.78 ^y^
Ground																	
With BA	0.64 ^e^	0.85 ^bc^	0.66	0.78 ^d^	0.71 ^c^	0.74 ^d^	0.82	0.78 ^d^	0.76 ^d^	0.74 ^c^	0.83	0.76 ^c^	0.74 ^c^	0.83	0.76	0.78	0.77
Without BA	0.60 ^f^	0.81 ^d^	0.63	0.74 ^d^	0.67 ^d^	0.73 ^d^	0.76	0.76 ^d^	0.73 ^d^	0.76 ^c^	0.82	0.72 ^d^	0.69 ^c^	0.78	0.74	0.74	0.73
Whole																	
With BA	0.69 ^d^	0.90 ^ab^	0.70	0.82 ^c^	0.74 ^c^	0.80 ^b^	0.84	0.83 ^c^	0.81^bc^	0.84 ^b^	0.86	0.80 ^b^	0.82 ^b^	0.89	0.80	0.83	0.85
Without BA	0.66 ^de^	0.88 ^b^	0.67	0.79 ^c^	0.71 ^c^	0.77 ^c^	0.82	0.82 ^c^	0.80 ^c^	0.82 ^b^	0.85	0.78^bc^	0.74 ^c^	0.85	0.78	0.80	0.81
Pooled SE	0.01	0.02	0.03	0.01	0.01	0.01	0.02	0.03	0.01	0.03	0.02	0.01	0.02	0.03	0.02	0.03	0.03
*p*-value																	
PS	0.001	0.001	0.012	0.027	0.034	0.021	0.027	0.030	0.021	0.003	0.453	0.001	0.002	0.521	0.029	0.043	0.023
DS	0.024	0.032	0.024	0.013	0.026	0.043	0.372	0.024	0.033	0.029	0.581	0.034	0.009	0.429	0.076	0.532	0.461
BA	0.035	0.061	0.072	0.427	0.531	0.376	0.348	0.381	0.412	0.735	0.672	0.523	0.281	0.217	0.521	0.631	0.562
PS × DS	0.044	0.043	0.375	0.031	0.381	0.537	0.334	0.432	0.287	0.854	0.924	0.638	0.643	0.587	0.634	0.824	0.349
PS × BA	0.035	0.522	0.411	0.290	0.627	0.624	0.357	0.540	0.254	0.697	0.836	0.724	0.251	0.359	0.648	0.761	0.427
DS × BA	0.062	0.374	0.50	0.251	0.343	0.597	0.450	0.723	0.528	0.589	0.738	0.536	0.358	0.687	0.731	0.538	0.721

^a–f^ Means without a common superscript within a column differ significantly (*p* < 0.05). ^x,y^ Means without a common superscript within a column differ significantly (*p* < 0.05). ^1^ Each value represents the mean of eight replicates (four birds per replicate).

**Table 6:** Effects of protein source (PS), diet structure (DS) and butyric acid (BA) supplementation on carcass characteristics ^1^ in broilers ^2^ at 36 days of age.

Effect	LW (g)	CW (g)	CY (%)	LQY (%)	BY (%)	ABF (%)
SBM						
Ground						
With BA	2366 ^ab^	1614 ^bc^	68.2 ^b^	31.0 ^b^	29.0 ^ab^	4.9 ^cd^
Without BA	2264 ^b^	1526 ^c^	67.4 ^b^	30.0 ^b^	28.0 ^bc^	5.1 ^c^
Whole						
With BA	2467 ^a^	1752 ^a^	71.0 ^a^	33.5 ^a^	31.8 ^a^	4.1 ^cd^
Without BA	2387 ^a^	1647 ^ab^	69.0 ^ab^	31.8 ^ab^	30.7 ^a^	4.6 ^cd^
CM						
Ground						
With BA	2040 ^d^	1316 ^d^	64.5 ^c^	27.3 ^d^	24.3 ^d^	5.3 ^b^
Without BA	1862 ^e^	1151 ^e^	61.8 ^d^	25.2 ^e^	21.2 ^e^	7.4 ^a^
Whole						
With BA	2208 ^c^	1497 ^cd^	67.8 ^b^	29.1 ^c^	27.2 ^c^	6.0 ^b^
Without BA	2131 ^cd^	1394 ^d^	65.4 ^c^	29.2 ^c^	26.5 ^c^	6.1 ^b^
Pooled SE	50.9	40.2	0.92	0.96	0.81	0.40
*p*-value						
PS	<0.001	<0.001	0.022	0.001	0.001	0.015
DS	0.015	<0.001	<0.001	0.004	0.001	0.045
BA	0.034	0.036	0.041	0.564	0.029	0.047
PS × DS	0.001	0.001	0.032	<0.001	0.041	0.410
PS × BA	0.041	0.012	0.854	0.720	0.037	0.078
DS × BA	0.032	0.024	0.037	0.045	0.030	0.043

^1^ LW= live weight, CW= carcass weight, CY (%) = carcass yield, LQY (%) = leg quarter yield, BY (%) = breast yield, ABF (%) = abdominal fat. ^a–e^ Means without a common superscript within a column differ significantly (*p* < 0.05). ^2^ Each value represents the mean of eight replicates (four birds per replicate).

## Data Availability

Not appliable.
